# X-Ray and CT Scan Based Prediction of Best Fit Tracheostomy Tube—A Pilot Study

**DOI:** 10.3390/diagnostics10080506

**Published:** 2020-07-22

**Authors:** Mel Corbett, Isobel Hughes, John O’Shea, Matthew G. Davey, Jane Savage, Joseph Hughes, Fintan Wallis

**Affiliations:** 1 Department of Radiology, University Hospital Limerick, V94F858 Limerick, Ireland; hughesis@tcd.ie (I.H.); M.DAVEY7@nuigalway.ie (M.G.D.); jane.savage1@hse.ie (J.S.); fintan.wallis@hse.ie (F.W.); 2 Department of Otolaryngology, University Hospital Limerick, V94858 Limerick, Ireland; jhughes@rcsi.ie; 3 Department of Anaesthesia and Critical Care, University Hospital Limerick, V94F858 Limerick, Ireland; johnoshea@alumnircsi.com

**Keywords:** tracheostomy, safety, X-ray, prediction

## Abstract

Tracheostomy is a commonly performed intervention in patients requiring ventilatory support. The insertion of inappropriately sized tracheostomy tubes carries a risk of decannulation, tissue damage, ventilatory difficulties, premature tube change or discomfort. Currently, no clear guidelines exist in determining the most appropriate size tube. Imaging of the airway preoperatively could aid clinical judgement and reduce risk. Patients included adult critical care patients who had appropriate preoperative imaging. The computed tomography scans and chest radiographs of patients were reviewed. Measurements of the airway were taken and scaled to the known internal diameter of an endotracheal tube. A four-point scoring system was developed to identify patients better suited to a non-standard sized tracheostomy tube. Data from 23 patients was analyzed using the Statistical Package for Social Sciences™ (SPSS). Four measured points on imaging corresponded to the patients’ appropriate tracheostomy tube size. Appropriate tracheostomy size correlates with tracheal diameter at endotracheal tube tip (r^2^ = 0.135), carina (r^2^ = 0.128), midpoint of larynx to carina (r^2^ = 0.146), bronchial diameter at the left mainstem (r^2^ = 0.323), and intrathoracic tracheal length (r^2^ = 0.23). Among our cohort, a score of 4 predicts the need for a larger tracheostomy tube. Simple imaging provides accurate measurement of patients’ airway dimensions. Our method ensures tube size is selected according to patient airway size, and potentially reduces the risks associated with inappropriate sizing.

## 1. Introduction

Tracheostomy is a commonly performed procedure in critical care patients [1]. Indications for tracheostomy include prolonged ventilatory support, upper airway obstruction or to aid patients with poor pulmonary clearance [2]. There are over 12,000 tracheostomies performed in the UK annually [3]. Both percutaneous and open surgical approaches are effective and safe methods of tracheostomy insertion and have comparable outcomes [3,4].

Tracheostomies are often performed in high risk patient populations and are associated with significant morbidity [5]. Minimizing the risks and complications associated with tracheostomy insertion has been highlighted as a priority in evidence based management of these patients [5].

Tracheostomy tubes are produced in a variety of materials, sizes and specifications by manufacturing companies. Minimizing risks to patients undergoing these procedures involves selecting the appropriate size tube tailored to a number of patient and disease factors. Standard tube sizes may be provided with some percutaneous tracheostomy insertion sets; however, these are not optimal for each individual patient [6]. Initial insertion of inappropriately sized tracheostomy tubes confers a number of risks, including decannulation, tissue damage, ventilatory difficulties, premature tube change or discomfort [7]. Currently there are no universally accepted guidelines on the correct size tracheostomy tube to insert for adult patients. Pandian et al. have examined the use of preoperative computed tomography (CT) scans to determine patient populations which may benefit from a non-standard tracheostomy tube [7]; however, the global availability of CT scanning is variable, depending upon healthcare resources [8]. The aim of our study was to demonstrate that preoperative imaging may provide valuable information that can be used to aid clinicians in tailoring tube size for their patient. 

## 2. Materials and Methods 

**Figure 1:**
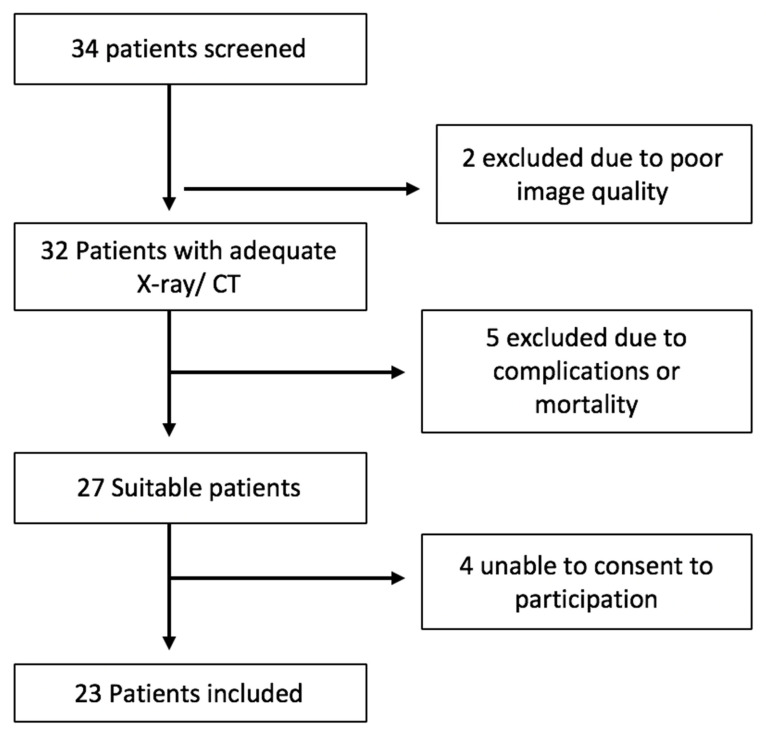
Inclusion criteria flow-chart.

Local institutional ethical approval was obtained. A retrospective observational cohort study was undertaken in our center. This included consecutive critical care patients diagnosed and treated in University Hospital Limerick, Ireland over a 12-month period between January 2018 and January 2019. We conducted a retrospective study of patient records and radiology images. Adult patients who had an appropriately sized tracheostomy at Limerick University Hospital were included in the study. Tracheostomies were performed using either a percutaneous or open surgical approach between the first and third tracheal cartilage rings. Patients were required to have a suitable quality chest X-ray or CT scan, in order to obtain airway measurements. All patients included were admitted to the critical care unit. All X-rays were taken using the Siemens Mobilette Mira Max machine, and window settings were adjusted to maximize contrast between soft tissue and trachea. Exclusion criteria included unsuitable imaging, patient mortality within 30 days of tracheostomy placement or complicated peri-operative course. Patients with poor quality radiological imaging unsuitable for scaled measurement were excluded from the study (Figure 1).

**Figure 2:**
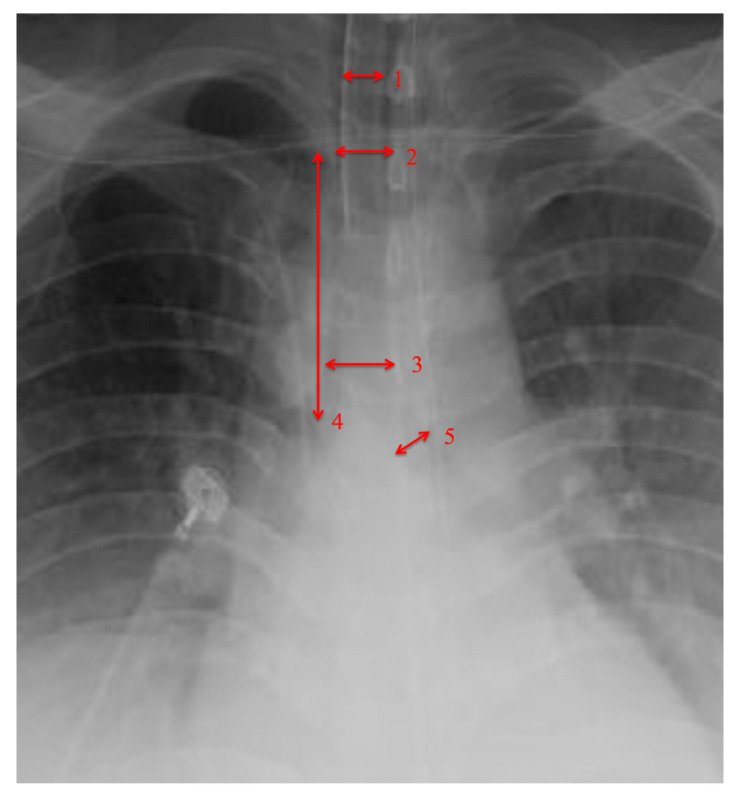
Measurement of trachea anatomy. 1 = endotracheal tube measurement for scale, 2 = trachea diameter at 1st rib, 3 = trachea diameter at carina, 4 = trachea length 1st rib to carina, 5 = left main bronchus diameter.

The airway dimensions of each patient were measured using chest radiographs or CT scans. When both imaging modalities were available, CT scans were compared to chest X-rays and X-ray measurements were accurate to 2 millimeters. We compared each patients’ airway measurements to appropriate tracheostomy tube outer diameter. Measured distances on chest X-rays were scaled using known measurements, such as internal diameter of endotracheal tube or tracheostomy tube. Measured airway dimensions were comparable to the published literature [9]. Measurements were taken of the diameter of the trachea at the 1st rib, diameter of the trachea at the carina, diameter of the left main bronchus and intrathoracic length of the trachea from the 1st rib to the carina (Figure 2).

Descriptive statistics were used for analysis of clinical patient information, data regarding co-morbities and previous intensive care admissions. Quantitative data was analyzed using Statistical Package for Social Sciences™ (SPSS™) version 26 (IBM SPSS Statistics, Armonk, NY, USA). Linear regression analysis was conducted. Variables found to correlate positively with need for non-standard tracheostomy tube size were incorporated into a four-point scoring system. The Spearmann rank correlation coefficient was used to determine correlation between tracheostomy outer cannula size and anatomical airway measurements.

## 3. Results

Twenty-three consecutive patients met the inclusion criteria for the study. Eleven patients were excluded based on the criteria or were not contactable to provide consent. There were 15 men and eight women included, with a median age of 63 years (range: 21–80 years) (Table 1). The majority of patients (19/23, 82.6%) underwent percutaneous tracheostomy insertion. 

All 23 patients had suitable chest radiographs, while two patients also had CT scans. Mean dimensions for tracheal diameter, intrathoracic tracheal length and left main bronchus diameter are illustrated in Table 2. Four measured points on imaging corresponded to the patients’ appropriate tracheostomy tube size (Table 3). Appropriate outer ring tracheostomy size were correlated with tracheal diameter at level of the first rib, where the rib reaches the sternum (r^2^ = 0.135); the carina (r^2^ = 0.128); bronchial diameter at the left mainstem (r^2^ = 0.323); and intrathoracic tracheal length from first rib to carina (r^2^ = 0.23). Among our cohort, having a tracheal diameter over 15 mm at both points, left main bronchus diameter of over 10 mm, and an intrathoracic tracheal length from first rib to carina of over 80 mm, predicts the need for a tube with an internal diameter larger than 8 cm (sensitivity = 100%, specificity = 88%). 

## 4. Discussion

Tracheostomies are frequently carried out on critically ill patients and carry an associated risk [10]. As there is no evidence-based guideline for sizing tracheostomy tubes, clinicians depend upon personal experience and availability when choosing a size for their patients. Subsequentially, patients are exposed to increased risk of complications should early tracheostomy change be indicated as a consequence of a poorly sized tube [11]. Ventilated patients with inappropriately small tubes may have difficulty with airway pressures [12]. While patients with tubes that are too large may suffer tracheal damage pain and tissue necrosis [13].

Plain film radiology of the thoracic cavity is indicated routinely in patients undergoing critical care management, and the application of information detailed from chest X-rays can be a cost-effective means of improving outcomes in patients undergoing tracheostomy. We acknowledge that CT evaluation of pre-tracheal skin and fat is valuable, in order to gauge the potential requirement for an extended-length tracheostomy tube, although there is limited availability of CT scanning in some healthcare scenarios [14]. Our analysis demonstrates that the application of simple imaging techniques may provide accurate measurement of anatomical airway dimensions. Our score provides a rapid, cost-effective and easily applicable stratification system to aid clinicians in sizing tracheostomy tubes, potentially reducing the risks associated with inappropriate sizing. Scaling tracheal measurements using known diameters of medical equipment, such as endotracheal tubes, allows for the accurate measurement of anatomical structures in a similar plane. The introduction of a radio-opaque measurable marker to facilitate easy prospective plain film thoracic analysis may be applied in many healthcare settings. Further prospective validation of this concept in clinical practice is required, in order to corroborate its value to tracheostomy outcomes. We advocate that the physician should appraise clinical and radiological information, and decide on the best fit tube based on each individual patient prior to undertaking a new tracheostomy.

While this pilot study supports the hypothesis that CXR may be used to identify patients requiring a non-standard tracheostomy tube size, larger cohort studies are needed. Larger cohorts would allow for the division of data into training and test datasets (which is not feasible in our cohort of 23 patients). We also appreciate that a CT evaluation will remain the gold standard for anatomical measurement when planning tracheostomy insertion.

**Figure 3:**
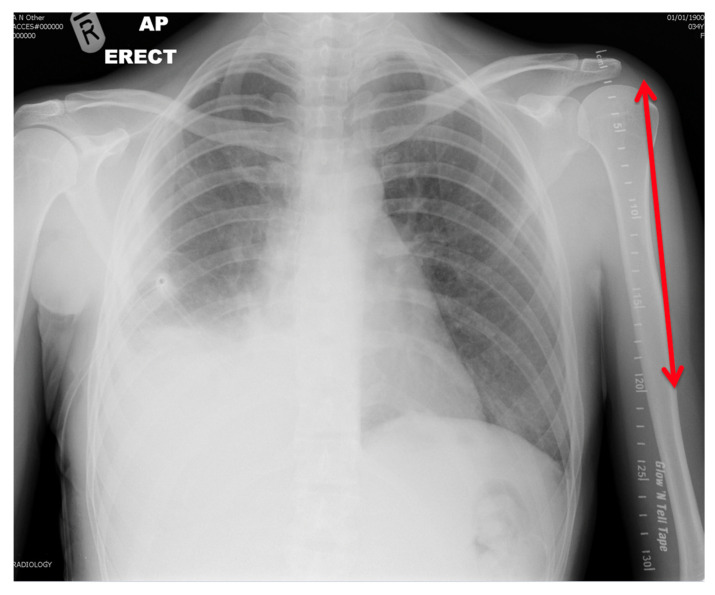
Radiograph demonstrating radio-opaque marker parallel to left humerus.

Adding a radio-opaque marker to plain film radiograph imaging of patient’s thoracic cavities provides an accurate means of increasing the information available prior to selecting the best sized tracheostomy tube for each patient (Figure 3). This may negate the need to use pre-existing devices to scale airway measurements, and allow for the inclusion of more patients prospectively. This information has the potential to improve patient safety, reduce complication rates and reduce the morbidity associated with new tracheostomy. This study demonstrates the potential advantages basic plain film imaging provides in the setting of tracheostomy insertion.

## Figures and Tables

**Table 1:** Demographics and tracheostomy type.

Characteristic	Total *n* = 23 *n*(%)
Sex-no. (%)	
Male	15(65)
Female	8(35)
Age-year	
Median (IQR)	63 (54–70)
Range	21–80
Technique-no. (%)	
Surgical	4(17)
Percutaneous	19(83)

**Table 2:** Airway dimensions.

Airway Measurement (mm)	Total *n* = 23 Mean (mm) (±SD)
Tracheal Diameter	15.75 (±4.03)
Intrathoracic Tracheal Length	82.09 (±20.82)
Left main bronchus	12.765 (±2.85)

**Table 3:** Predictors of a larger than standard tracheostomy tube using chest plain film radiographs.

• Tracheal Diameter Measured at 1st Rib > 15 mm
• Tracheal diameter measured at carina > 15 mm
• Intrathoracic Tracheal length from 1st rib to carina > 80 mm
• Left main-stem bronchus diameter > 10 mm
